# Label-free photoacoustic and ultrasound imaging for murine atherosclerosis characterization

**DOI:** 10.1063/1.5142728

**Published:** 2020-04-03

**Authors:** Gurneet S. Sangha, Craig J. Goergen

**Affiliations:** 1 Weldon School of Biomedical Engineering, Purdue University, 206 S. Martin Jischke Dr., West Lafayette, Indiana 47907, USA; 2 Center for Cancer Research, Purdue University, 201 S. University St., West Lafayette, Indiana 47907, USA

## Abstract

Dual-modality photoacoustic tomography (PAT) and 4D ultrasound (4DUS) imaging have shown promise for cardiovascular applications, but their use in murine atherosclerosis imaging is limited. This study used PAT and 4DUS to correlate altered arterial strain and hemodynamics to morphological changes and lipid localization in a murine partial carotid ligation (PCL) model of atherosclerosis. Validation experiments showed a positive correlation between the PAT signal-to-noise ratio and plaque lipid composition obtained from oil-red O histology. Cross-sectional *in situ* PAT and longitudinal *in vivo* ultrasound imaging was performed using a 40 MHz transducer. Ultrasound timepoints included days 0, 1, 4, 7, 10, and 14 for hemodynamic and strain assessment, and 1100 nm and 1210 nm PAT was implemented at the study end point for hemoglobin and lipid characterization. These study groups were then separated into day 4 post-PCL with (n = 5) and without (n = 6) Western diet feeding, as well as days 7 (n = 8), 10 (n = 8), and 14 (n = 8) post-PCL, in addition to a sham control group on a Western diet (n = 5). Overall, our data revealed a substantial decrease in left carotid artery pulsatility by day 7. The hemodynamic results suggested greater disturbed flow in the caudal regions resulting in earlier vessel stenosis and greater lipid deposition than cranial regions. Morphological and compositional data revealed heterogeneous vascular remodeling between days 0 and 7, with a rapid decrease in the vessel volume/length and the presence of both intraplaque hematoma and lipid deposition at day 10 post-PCL. These results highlight the utility of utilizing dual-modality PAT and 4DUS to study atherosclerosis progression.

## I. INTRODUCTION

Development of dual-modality photoacoustic tomography (PAT) and ultrasound imaging is an emerging area of biomedical research, as these complementary modalities allow for advanced visual and quantitative interpretation for a plethora of multifaceted diseases. Ultrasound uses high-frequency sound waves to measure the acoustic impedance between tissue boundaries, allowing the user to obtain a variety of information regarding tissue morphology and hemodynamics. Conversely, PAT utilizes nanosecond pulsed laser light to thermoelastically induce acoustic waves in order to obtain spatially relevant compositional images (i.e., blood and lipid localization^1^). The unique characteristics of these imaging techniques have the potential to improve our understanding of the world's most life-threatening diseases.

Cardiovascular disease, in particular, remains one of the leading causes of death globally with atherosclerosis resulting in a variety of complications ranging from impaired lower-limb mobility to ischemic myocardial infarction and strokes.^2^ Current cardiovascular applications of dual-modality PAT and ultrasound are primarily focused on diagnosis of atherosclerosis,^3–9^ identification of thrombus,^10,11^ or ablation of cardiac arrhythmias.^12–14^ Developmental efforts in intravascular photoacoustic imaging have especially shown potential to quantify plaque burden in the hopes to identify rupture-prone vulnerable plaques vs benign stable plaques.^15,16^ Moreover, recent advances in 4D ultrasound (4DUS) imaging have opened opportunities to improve kinematic characterization of cardiac^17^ and vascular^18^ tissues. Dynamic volumetric information provided by 4DUS can be used to estimate 3D Green-Lagrange strain using a direct deformation estimation (DDE) method.^19^ One under-investigated area of research, however, is consideration of how these novel techniques can be used to better understand atherosclerosis progression.

We demonstrated the capability of using dual-modality PAT and ultrasound to characterize atherosclerosis progression in a small animal model. We first performed *ex vivo* validation studies using PAT to quantify lipid-burden in murine atherosclerotic plaques. We then performed longitudinal *in vivo* ultrasound and cross-sectional *in situ* PAT studies to investigate the interplay between vessel hemodynamics and mechanics on atherosclerosis progression. Standard ultrasound was used to quantify changes in hemodynamics, 4DUS to quantify altered kinematics, and PAT to assess hemoglobin (blood) and lipid localization throughout the carotid artery. Overall, we evaluated the hypothesis that vascular regions of disturbed flow and decreased vessel pulsatility are more susceptible to lipid deposition during plaque formation.

## II. RESULTS

### A. PAT resolution characterization

**FIG. 1.**
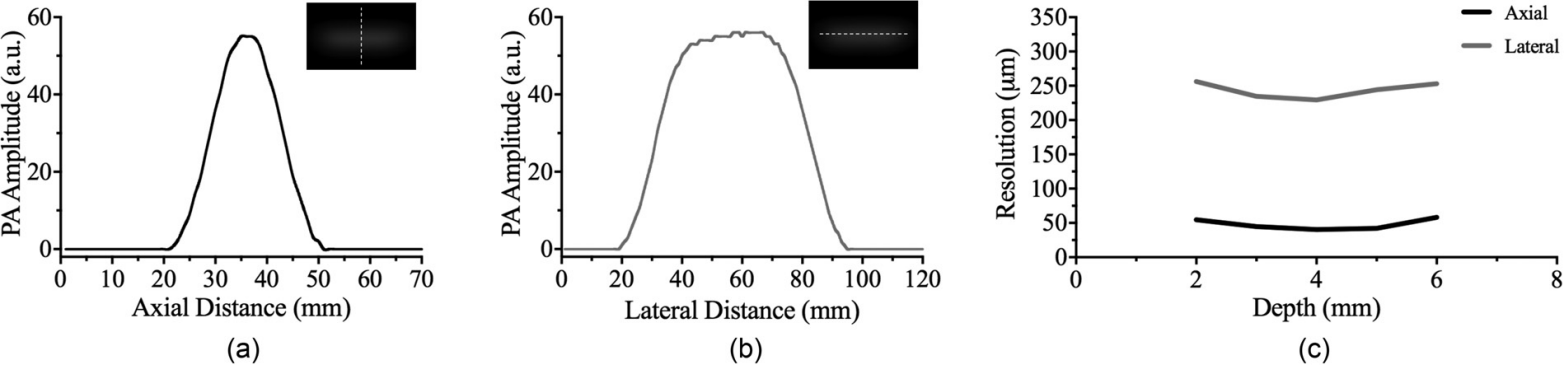
Axial (a) and lateral (b) profile of PAT signal amplitude using 20% PVA with five embedded 50 *μ*m tungsten wires. Depth profiling resolution measurements with 1210 nm light revealed an axial resolution of 48 ± 8 *μ*m and a lateral resolution of 243 ± 11 *μ*m (c). The insets in (a) and (b) show PAT images of tungsten wire and profile measurement location (white dotted line).

Spatial resolution was characterized using five 50 *μ*m tungsten wires that were inserted into 20% tissue-mimicking PVA. Figures 1(a) and 1(b) shows the PAT images of these wires, as well as the PAT signal amplitude along the axial and lateral directions. Our results showed small depth-wise variation in resolution with an axial resolution of 48 ± 8 *μ*m and a lateral resolution of 243 ± 11 *μ*m [Fig. 1(c)].

### B. *Ex vivo* carotid artery lipid burden results

**FIG. 2.**
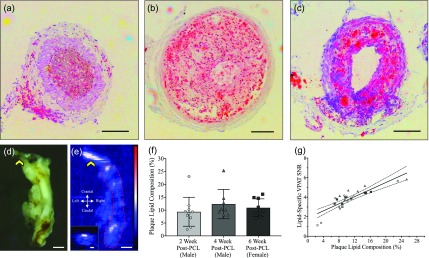
Validation of PAT to quantify lipid burden in a murine model of atherosclerosis. Lipid accumulation in mice that underwent a PCL varied greatly between animals as shown by ORO stained histology [(a)–(c)]. Comparison of gross (d) and PAT (e) LCA (red dotted line) showed adequate spatial correlation of the lipid signal. Suture is highlighted by a yellow arrow. Plaque lipid composition did not vary significantly between 2 and 6 ± 1.7 weeks post-PCL procedure regardless of sex (f). Linear regression statistics of plaque lipid composition vs lipid specific PAT SNR showed positive correlation (R^2^ = 0.72, n = 27, *p *<* *0.001; (g) scale bar (a)–(c), inset (e): 100 *μ*m and (e): 1 mm.

Qualitative assessment of ORO stained LCA revealed a diverse spectrum of vessel morphology in mice two weeks post-PCL. The murine plaque phenotype ranged from fibrotic plaques with visible wall thickening [Fig. 2(a)] to complete occlusion with lipid accumulation through the lesion [Fig. 2(b)] or severe lipid accumulation without complete occlusion of the carotid artery [Fig. 2(c)]. Quantitative assessment of PAT images [Figs. 2(d) and 2(e)] showed that on average all three murine groups formed plaques with similar lipid dense regions regardless of sex and post-PCL euthanasia [Fig. 2(f)]. Particularly, we observed 9.4 ± 5.6% lipid composition in 2-week post-PCL male mice, 12.4 ± 5.7% lipid composition in 4-week post-PCL male mice, and 11.0 ± 3.5% lipid composition in 6 ± 1.7 week post-PCL female mice. These results also showed considerable variation in lipid dense regions and, as a result, no statistical significance among the three experimental groups (*p *>* *0.05). We then assessed our capability to use PAT SNR to quantify lipid dense regions in the plaque-rich LCA. We found that linear regression statistics resulted in a R^2^ value of 0.73 when using 27 spatially correlated short-axis PAT and ORO histological sections [*p *<* *0.001; Fig. 2(g)].

### C. Hemodynamic and kinematic characterization results

**FIG. 3.**
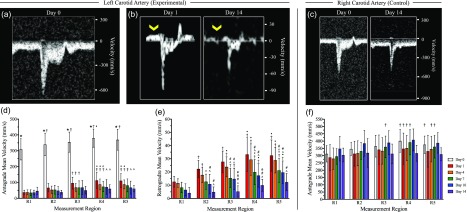
Hemodynamic characterization of LCA mean velocities between day 0 (a) and days 1 through 14 post-PCL (b) revealed a statistically significant decrease in antegrade blood flow (d) and an increase in retrograde blood flow (e). Caudal regions (R4 and R5) had statistically greater antegrade and retrograde velocities compared to cranial regions (R1 and R2) until day 7, as well as a steady decrease in overall velocity until day 14. Conversely, the contralateral control RCA revealed healthy pulsed wave Doppler (c) and antegrade flow velocities (f) over 14 days. Statistical significance: *p *<* *0.05 (★ = significance compared to all other days in the same region, † = significance compared to the same day in region 1, • = significance compared to the same day in region 2, ∧ = significance compared to day 1 in the same region, # = significance compared to day 4 in the same region, & = significance compared to day 7 in the same region, and ¥ = significance compared to day 10 in the same region).

Hemodynamic assessment of pulsed wave Doppler images revealed initiation of disturbed flow in the LCA [Figs. 3(a) and 3(b)] and maintenance of laminar flow in the RCA post-PCL [Fig. 3(c)]. Quantification of LCA velocities also confirmed disturbed flows with a 76% average decrease in mean antegrade velocities [Fig. 3(d)] and an overall increase in retrograde velocities [Fig. 3(e)] post-PCL. Regional assessment of both antegrade and retrograde velocities showed statistically greater velocities in the caudal (R4 and R5) compared to the cranial (R1 and R2) regions (*p *<* *0.05) until day 7, and an overall significant decrease in mean velocities between days 1 and 14 post-PCL (*p *<* *0.05). The RCA revealed a slight decrease in mean velocities between day 0 and days 1 and 4 due to the surgical procedure. We also observed an increase in mean velocities on days 7 and 10, followed by a subsequent decrease in mean velocity on day 14 [Fig. 3(f)]. Peak velocity measurements showed similar trends to the mean velocity data, as found in supplementary material, Fig. S2. The sham hemodynamic data for both carotid arteries followed a similar trend to the experimental RCA results (supplementary material, Fig. S3).

**FIG. 4.**
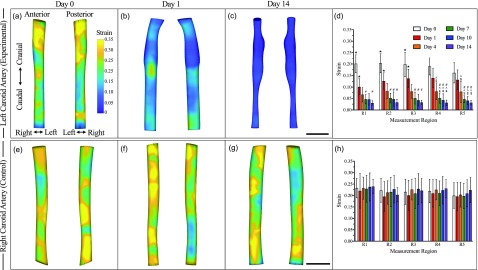
Representative 3D images of maximum first-principal Green-Lagrange strain in the LCA post-PCL [(a)–(c)] and the contralateral control RCA [(e)–(g)]. Regional assessment of strain in the LCA (d) suggested a decrease in vessel pulsatility after suture placement at day 1 followed by a steady decrease until day 7. Strain measurements in the RCA suggested healthy vessel pulsatility (h). Strain values were overlaid on segmented volumes for visualization. Statistical significance determined at *p *<* *0.05 (★ = significance compared to all other days in the same region, $= significance compared to day 0 in the same region, ∧ = significance compared to day 1 in the same region, and # = significance compared to day 4 in the same region). Scale bar: 1 mm.

Strain characterization using the DDE method revealed heterogeneous pulsatility in both the left [Figs. 4(a)–4(d)] and right [Figs. 4(e)–4(h)] carotid arteries. Regional assessment of LCA strain showed a rapid decrease in strain during the 14-day study [Fig. 4(d)] with a large reduction in strain from day 0 (19.1 ± 4.5%) to days 1 (12.7 ± 4.1%), 4 (7.8 ± 3.6%), and 7 (4.9 ± 2.1%). The RCA strain values, however, maintained vessel pulsatility throughout the study [day 1: 21.8 ± 5.2%; day 14: 22.6 ± 4.3%; Fig. 4(h)]. Comparison of day 4 post-PCL strain results with and without Western diet feeding showed no statistical difference in vessel pulsatility (supplementary material, Fig. S4). Interestingly, the sham strain data indicated a decrease in vessel pulsatility post-PCL, followed by return to healthy values by day 10 (supplementary material, Fig. S3). A comparison of strain values using the DDE method and M-mode analysis revealed similar pulsatility trends in both carotid arteries (supplementary material, Fig. S5).

### D. Morphological and compositional characterization results

**FIG. 5.**
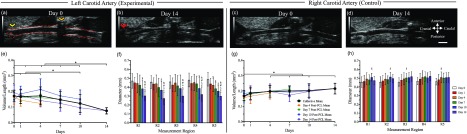
Morphological characterization of LCA between days 0 (a) and 14 (b) suggested a heterogeneous remodeling response due to PCL until day 7, followed by rapid reduction of both volume/length (e) and diameter (f). Moreover, a statistically significant increase in RCA volume/length (g) and increase in diameter (h) from day 0 (c) through day 14 (d) suggested compensatory remodeling due to LCA stenosis. Statistical significance determined at *p *<* *0.05 (* = significance between groups, $= significance compared to day 0 in the same region, ∧ = significance compared to day 1 in the same region, # = significance compared to day 4 in the same region, & = significance compared to day 7 in the same region, and ¥ = significance compared to day 10 in the same region). Scale bar: 1 mm.

Morphological analysis indicated interesting vessel remodeling in both the left [Figs. 5(a) and 5(b)] and right [Figs. 5(c) and 5(d)] carotid arteries. Diameter measurements suggested no changes in vessel morphology between days 0 and 4 [Fig. 5(f)]. Volume/length measurements, however, clearly showed dynamic changes in the LCA size between days 1 and 4 with some animals experiencing an increase in volume/length and others experiencing a decrease in volume/length [Fig. 5(e)]. At day 10, we observed a 24.7% decrease in volume/length and at day 14, we find a 53.6% decrease in volume/length compared to day 4 (*p *<* *0.05). Interestingly, the diameter measurements revealed statistically significant stenosis in region 5 by day 4 (*p *<* *0.05), while regions 1–4 showed a statistically significant decrease in diameter much later at day 10 (*p *<* *0.05). Compensatory effects of RCA due to LCA stenosis were seen in volume/length values as early as day 4 [Fig. 5(g); *p *<* *0.05]. The RCA also showed signs of gradual diameter increase [Fig. 5(h)] between days 0 (0.46 ± 0.05 mm) and 14 (0.51 ± 0.05 mm). Sham morphological data showed no statistically significant changes in the vessel diameter or volume/length (supplementary material, Fig. S3). Volume data have been provided in supplementary material, Fig. S6.

**FIG. 6.**
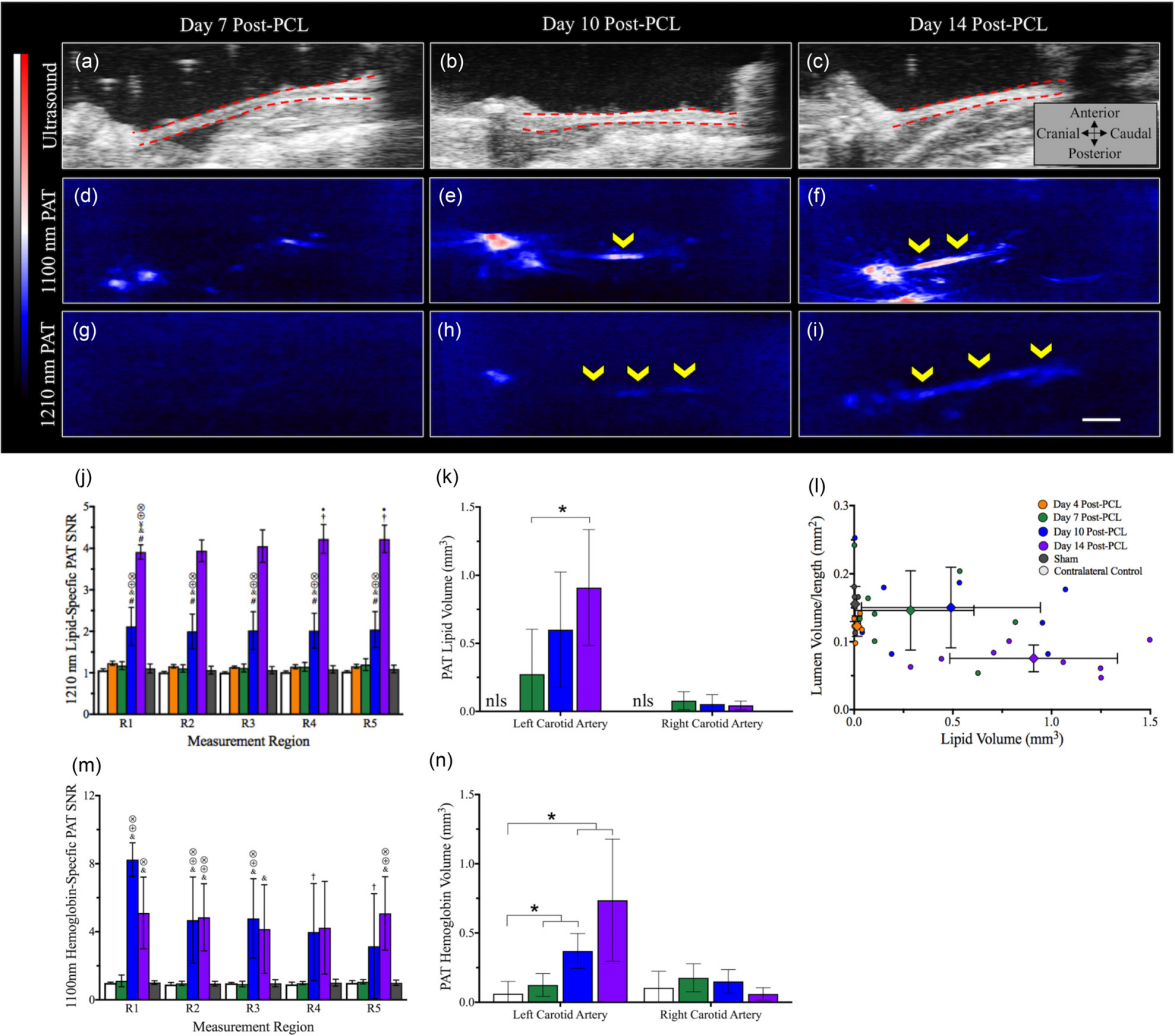
Representative *in situ* ultrasound [(a)–(c)], 1100 nm PAT [(d)–(f)], and 1210 nm PAT [(g)–(i)] images of LCA at days 7, 10, and 14 post-PCL. Regional assessment of lipid specific PAT SNR (j) and PAT lipid volume (k) suggested statistically significant and rapid lipid accumulation between days 7 and 14, with greater lipid accumulation in the caudal most regions at day 14. Comparison of the lipid volume and lumen volume/length suggests compensatory remodeling of the LCA until day 10, potentially due to Glagov remodeling, followed by a rapid decrease in the lumen volume and increase in lipid volume (l). Regional assessment of hemoglobin specific PAT SNR (m) and PAT hemoglobin volume (n) revealed the presence of intraplaque hemorrhages at day 10 with a 98.9% increase blood volume between days 10 and 14. Statistical significance determined at *p *<* *0.05 (* = significance between groups, † = significance compared to the same day in region 1, # = significance compared to day 4 in the same region, & = significance compared to day 7 in the same region, ⊗ = significance compared to sham group, ⊕ = significance compared to right contralateral control RCA). Scale bar: 1 mm.

Hemoglobin PAT contrast revealed regions of blood accumulation at days 10 and 14 [Figs. 6(a)–6(f)]. We observed rapid, yet variable, hemoglobin accumulation between days 7 and 10 post-PCL [*p *<* *0.05; Fig. 6(m)], especially in the cranial most region. Hemoglobin volume data revealed a 98.9% increase in blood accumulation between days 10 and 14 [Fig. 6(n)]. PAT lipid deposition and volume measurement revealed similar trends as the morphological data. We observed a statistically significant increase in lipid deposition, as quantified by 1210 nm SNR, at day 10 along the carotid artery [Figs. 6(g)–6(i)]. Interestingly, two out of eight animals showed the presence of lipid accumulation at day 7. We also observed a 131.5% increase in lipid-specific SNR between days 10 and 14 with statistically greater lipid deposition (*p < *0.05) in the caudal region (R4 and R5) compared to the cranial region [R1 and R2; Fig. 6(j)]. The day 4, sham, and contralateral control carotid arteries showed minimal signs of lipid or hemoglobin accumulation. Lipid volume data indicated a similar trend with a statistically significant increase (*p < *0.001) in lipid volume between days 7 (0.27 ± 0.33 mm^3^) and 14 [0.91 ± 0.42 mm^3^; Fig. 6(k)]. Additionally, we observed an increase in lipid accumulation between days 7 and 10 but maintenance of lumen volume/length followed by a substantial decrease in day 14 volume/length [Fig. 6(l)]. Hemoglobin and lipid deposition graphs for individual groups have been provided in supplementary material, Figs. S7 and S8.

### E. Histology

**FIG. 7.**
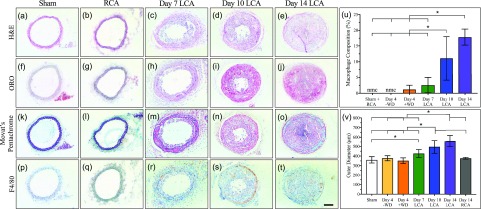
Histology and macrophage immunohistochemistry of day 7, 10, and 14 post-PCL and sham groups, as well as contralateral control RCA. Staining consisted of H&E [(a)–(e)], ORO [(f)–(j)], Movat's pentachrome [(k)–(o)]. Macrophage infiltration was assessed with F4/80 immunohistochemistry [(p)–(t)] and revealed rapid inflammation between days 4 and 10 (u). LCA measurements showed a 46.1% greater outer wall diameter in day 14 post-PCL compared to sham mice (v). Statistical significance determined at *p *<* *0.05. Scale bar: 100 *μ*m.

Histological assessment of the LCA confirmed significant vessel remodeling during the first two weeks post-PCL. H&E staining revealed LCA stenosis by day 10 for all experimental animals [Figs. 7(a)–7(e)] and at day 7 in two out of the eight experimental animals [Fig. 7(c)]. The ORO histology verified the rapid LCA lipid accumulation between days 7 and 10 [Figs. 7(f)–7(j)]. Interestingly, Movat's pentachrome confirmed the presence of neovascular formation by day 10 post-PCL [Figs. 7(k)–7(o)], as shown by red muscle staining around vessel-like structures. Magnified images of histology sections can be found in supplementary material, Fig. S10. Finally, F4/80 immunohistochemistry [Fig. 7(p)–7(t)] suggested inflammatory response initiation between days 4 and 7 followed by a slight increase in macrophage infiltration between days 7 and 14. Quantitative analysis of our F4/80 histology suggests percent macrophage compositions of 0% in the sham group and day 4 post-PCL on a normal chow diet, 1.2 ± 1.4% in day 4 post-PCL with Western diet mice, 2.6 ± 2.4% in day 7 post-PCL mice, 11 ± 6.9% in day 10 post-PCL mice, and 17.8 ± 2.6% in day 14 post-PCL mice [Fig. 7(u)]. Further analysis of arterial cross section revealed diameter measurement of 377.6 ± 26.5 *μ*m at day 4 post-PCL on a normal chow diet and 352.1 ± 29.5 *μ*m at day 4 post-PCL, 426.8 ± 41.2 *μ*m at day 7 post-PCL, 496.5 ± 67.6 *μ*m at day 10 post-PCL, and 543.6 ± 72.3 *μ*m at day 14 post-PCL, all from animals on a Western diet [Fig. 7(v)]. The sham and contralateral control RCA histology revealed vessel diameters of 360.6 ± 32.8 *μ*m and 377.8 ± 9.7 *μ*m, respectively, with patent vessels and no signs of inflammation.

## III. DISCUSSION

We present here a dual-modality PAT and ultrasound approach to study disease progression in a surgically induced murine model of atherosclerosis. Numerous mouse models have been developed to mimic various aspects of atherosclerosis pathology, including Western diet feeding,^20^ perivascular carotid cuff placement,^21^ complete carotid artery ligation,^22,23^ and PCL^24^ in atheroprone mice. For this study, we utilized ApoE KO mice in combination with the PCL procedure to create disturbed flow throughout the carotid artery that leads to rapid and measurable plaque accumulation.^24–27^ The presence of decreased antegrade flow and increased retrograde flow also allows for a general measure of laminar and oscillatory shear stress. Moreover, histological assessment of these atherosclerotic vessels reveals diverse plaque morphology with regions of complete stenosis, but limited lipid accretion and other partially stenosed lipid dense regions (Fig. 2). Indeed, surgical induction of atherosclerosis using the PCL provides a robust model to assess how hemodynamics and kinematics impact vessel morphology and lipid deposition.

Current applications for murine PAT imaging of atherosclerosis are limited, as the literature has primarily focused on developing this technique for intravascular assessment of plaque stability in humans. Therefore, we provide evidence that it is possible to nondestructively assess the lipid composition of murine plaques by characterizing the resolution of our PAT system and performing quantitative *ex vivo* validation experiments. The resolution characterization of our PAT system was better in the axial direction (48 ± 8 *μ*m) than in the lateral direction (243 ± 11 *μ*m). All 3D PAT images were, therefore, acquired in the long-axis plane to take advantage of the better axial PAT resolution. Our *ex vivo* validation study provides further evidence of quantifying lipid burden as the PAT SNR and plaque lipid composition have a positive correlation with R^2^ of 0.72 (*p < *0.001). These results also suggest that all PCL-induced carotid plaques have comparable lipid accumulation 2 weeks post-procedure regardless of sex and euthanasia timepoint post-PCL. Together, these validation experiments provide strong evidence for the use of PAT to quantify lipid burden in small animals.

Maximum first-principal Green-Lagrange strain values obtained from 4DUS datasets using a DDE method were compared to conventional M-mode analysis of circumferential cyclic strain. Our circumferential cyclic strain values for both LCA and RCA were comparable to previously published values of approximately 25% in healthy carotid arteries.^28^ Additionally, our maximum first-principal strain and circumferential cyclic strain values showed similar trends with both revealing a rapid decrease in vessel pulsatility between days 0 and 7 (supplementary material, Fig. S5). Our maximum first-principal strain values, however, were slightly lower than the circumferential cyclic strain data, possibly due to averaging between the more pulsatile anterior walls with the less pulsatile posterior walls that occurs when M-mode images are used to estimate average circumferential strain.

Hemodynamic and kinematic characterization revealed abrupt changes in LCA forces as early as day 1 post-PCL. Mean velocity measurements show a 76% decrease in antegrade flow, suggesting initiation of low shear stress and an increase in both retrograde flow and oscillatory shear stress. The manifestation of these altered forces, in combination with a suture-induced decrease in vessel pulsatility, may help explain the rapid atherosclerosis progression we observed in this model. Regional assessment of LCA hemodynamics revealed statistically greater antegrade and retrograde velocity (*p < *0.05) in the caudal regions (R4 and R5) compared to the cranial regions (R1 and R2). These data suggest greater disturbed flow patterns in the caudal carotid, possibly leading to more significant lipid deposition.^29^

According to Bernoulli's principle, as a vessel diameter decreases, the blood flow velocity should increase. Therefore, another noteworthy observation was a significant decrease in antegrade and retrograde velocity throughout the study (*p *<* *0.05). Qualitative assessment in color Doppler images suggests increased blood flow near the LCA bifurcation by day 7 post-PCL (data not shown), suggesting increased collateral artery formation or dilation of the superior thyroid artery. Additionally, our 1100 nm PAT images and Movat's pentachrome histology provided evidence for neovascular formation throughout the plaque. Day 10 hemoglobin-specific PAT contrast also suggests greater blood vessel formation toward the cranial regions compared to the caudal region. We suspect these regions to be intraplaque hemorrhages based on the hemoglobin-specific optical contrast we observed post-saline perfusion. These intraplaque hemorrhages can also be visualized through gross dissection images (supplementary material, Fig. S1). Overall, our results suggest that blood flow velocity decreases in the LCA, possibly due to compensatory RCA remodeling and/or collateral and neovascular formation within and surrounding the LCA.

Morphological and lipid data provide additional supportive evidence to suggest that caudal regions experience more aggressive atherosclerosis progression due to greater disturbed blood flow. Diameter measurements in caudal region 5 revealed significant stenosis as early as day 4 compared to significant stenosis by day 10 in regions 1–4. Moreover, our lipid deposition results show a statistically greater lipid accumulation in the caudal region (R4 and R5) compared to the cranial regions (R1 and R2) on day 14. This is especially interesting as our volume/length data show a dynamic change in vessel size between days 0 and 4, followed by significant stenosis on days 10 and 14 where we also observe the presence of a robust lipid signal (*p < *0.05; Fig. 5). We also observed lipid contrast through PAT in two out of eight day 7 post-PCL animals, which was confirmed with H&E and ORO histology. Analysis of lipid volume vs lumen volume/length provides evidence for Glagov remodeling as the vessel resists stenosis between days 4 and 10, followed by a rapid decrease in lumen volume/length by day 14 (Fig. 6).^30–32^ Quantitative histological analysis revealed a 46.1% greater LCA outer diameter in day 14 post-PCL compared to sham animals. Previous work in humans has shown that approximately 60% of large elastic arteries experience Glagov remodeling,^32,33^ which may explain the large variability in our murine study.

We also quantified contralateral control RCA metrics and compared them to the experimental LCA. Overall, we observed maintenance of healthy pulsatility and increased volume/length (*p < *0.05) and vessel diameter values, possibly due to compensatory effects for decreased flow through the LCA. Interestingly, the significant increase in RCA volume/length as early as day 4, before LCA stenosis was observed, may suggest a preparatory remodeling response due to decreased flow through the LCA. Sham animals did not show an increase in carotid volume/length, thus confirming that this change is not attributed to natural animal growth. Furthermore, we observed a decrease in RCA velocity at days 1 and 4 post-PCL as the animals recovered from the procedure (supplementary material, Fig. S9). The velocity in the RCA returned by day 7, but decreases again by 19% between days 10 and 14 post-PCL. The sham group shows a similar response, suggesting that the Western diet may have systemic effects on cardiovascular hemodynamics.^34,35^

We also investigated the use of strain as a predictor of atherosclerosis induction. We observed a significant decrease in vessel pulsatility at day 1 post-PCL and a second substantial decrease in the strain at day 4 post-PCL [Fig. 4(d), *p *<* *0.05]. This decrease in strain may be due to the initiation of atherosclerosis progression, as marked by macrophage infiltration and early lipid deposition. Therefore, a separate subset of animals was imaged until day 4 post-PCL with (n = 5) and without (n = 6) Western diet feeding. We did not observe a significant difference in regional strain between these groups, suggesting that acute changes in vessel pulsatility are mainly due to the suture placement and not a diet, similar to what has been previously shown in the iliac artery.^36^ H&E, ORO, and F4/80 staining also confirmed a lack of inflammation and subsequent plaque formation from these mice at day 4, providing further evidence for the dominant role of altered hemodynamics.

This study also highlights several limitations that may inspire future work. The advantage of *in situ* PAT is that the majority of the light is delivered to the tissue of interest; thus, calibration methods are not required to account for optical properties of more superficial endogenous absorbers. The disadvantage, however, is that the lack of longitudinal *in vivo* imaging limits lesion characterization. Moreover, the lack of intraluminal pressure when the animal is alive makes it challenging to correlate heterogeneous 4DUS strain data with lipid deposition to assess differences in lipid accumulation in the anterior and posterior walls. Future work will, therefore, be needed to identify the optimal illumination geometry and transducer bandwidth for lipid-specific imaging deep within tissue.^37–39^

In summary, while previous research has utilized advanced imaging techniques to study the multifaceted nature of atherosclerosis pathology,^20,40–43^ limited work has been done using PAT to correlate regional changes in hemodynamics and kinematics to lipid deposition and vascular remodeling. The work highlighted here provides evidence of a positive correlation between lipid composition within plaque and lipid-specific PAT SNR using a small animal model of atherosclerosis. We also present advanced methods using 4DUS to quantify morphological and kinematic changes during disease development. Overall, a dual-modality PAT and ultrasound approach to elucidate the complex nature of atherosclerosis development is a promising strategy that may eventually provide both research and clinical benefits.

## IV. CONCLUSIONS

The data presented here provide proof-of-concept evidence for the use of PAT to nondestructively image atherosclerosis. Strain analysis revealed spatially heterogeneous pulsatility throughout both the LCA and RCA. Morphological and lipid analysis was suggestive of murine Glagov remodeling with diverse vessel remodeling between days 0 and 7, followed by a rapid decrease in the vessel size after day 10. Hemoglobin-specific PAT contrast also suggested intraplaque hematoma formation as early as day 10. These results revealed that regions of greater oscillatory flows experience more aggressive lipid deposition and earlier vascular remodeling than regions of more unidirectional flows. Taken together, these results highlight the potential of utilizing dual-modality PAT and 4DUS to characterize and study plaque progression in small animal models, while also providing a starting point for future clinical studies aimed at assessing human atherosclerosis.

## V. METHODS

### A. Photoacoustic tomography system specifications

Our dual-modality PAT and ultrasound system consisted of a Nd:YAG pulsed optical parametric oscillator laser (Surelite EX, Continuum) coupled with a high-frequency small animal ultrasound system (Vevo2100, FUJIFILM VisualSonics). The Nd:YAG laser produced 5 ns pulses at 10 Hz ranging from 670 to 2500 nm that was delivered to the tissue of interest using a bifurcated fiber optic bundle. Our 2 m fiber optic bundle contained a 1.0 cm opening diameter and 18 mm × 2 mm rectangular terminals, producing an optical fluence of 40 mJ/cm^2^. The resulting acoustic waves were captured using a 40 MHz (MS550D) center frequency transducer that consisted of 256 piezoelectric element arrays. The laser and ultrasound were coupled using a pulse generator (9200, Quantum Composers) that sent appropriately timed 10 Hz, 5 V inverted signals to both the q-switch and flashlamp, as well as a normal 10 Hz, 5 V pulse signal to the ultrasound system.

### B. PAT resolution characterization

A depth-profiling phantom was fabricated to characterize the resolution of our PAT system. This phantom consisted of tissue-mimicking 20% polyvinyl alcohol (PVA) phantom^44^ with five embedded 50 *μ*m tungsten wires^45–47^ spaced 1 mm from one another. PVA was prepared using a method described previously.^48^ The phantom was then imaged using 1210 nm PAT with five grayscale images collected with the transducer focused on individual tungsten wires. The PAT gain was optimized to minimize beamforming artifacts due to over absorption of light. MATLAB was then used to perform plot-profile analysis^1^ to calculate the full width at half maximum (FWHM) in both the axial and lateral directions (Fig. 1). The axial and lateral resolutions were then calculated using the FWHM and the known tungsten wire diameter [D_t_; Eq. (1)]^49^

$$\text{Resolution}=\sqrt{{\left(\text{FWHM}\right)}^{2}-{\left(D_{t}\right)}^{2}}.$$ (1)

### C. Small animal atherosclerosis induction

Murine atherosclerosis was induced using the partial carotid ligation (PCL) procedure on apolipoprotein E-deficient (ApoE KO) mice (supplementary material, Fig. S1).^24^ ApoE KO mice were obtained from Jackson Laboratory (Bar Harbor, ME) and fed a standard chow diet until the time of surgery. All animals were anesthetized using a small animal anesthesia system (SomnoSuite, Kent Scientific) using 2%–3% isoflurane and 225 ml/min room air,^50^ and vital signs were closely monitored to ensure optimal anesthetic induction. The aseptic technique was used to sterilize the animal prior to exposing the left carotid artery (LCA) and ligating the internal, external, and occipital arteries. The superior thyroid artery was left open, inducing disturbed flow characterized by both low and oscillatory shear stress.^24^ The incision was then closed using 7–0 prolene sutures and buprenorphine was subcutaneously injected at a dose of 0.05–0.1 mg/ml. Finally, all animals were placed on a Western diet (TD.88137, Envigo) consisting of 42.7% carbohydrates, 42% fat, and 15.2% protein. Pulsed wave Doppler was used to confirm disturbed biphasic flow, and 4DUS and color Doppler were used to verify arterial ligation. The Purdue Animal Care and Use Committee approved all of the outlined animal procedures and experiments (Protocol ID:1302000818).

### D. PAT plaque lipid burden quantification validation

#### 1. *Ex vivo* common carotid artery image acquisition

Initial *ex vivo* characterization on atherosclerotic carotid arteries was performed to assess the capability of our PAT system to identify and quantify lipid burden. Table I contains a summary of all animal groups and imaging timepoints. Atherosclerotic plaques were induced via the PCL procedure, followed by euthanasia and excision of the LCA and supporting tissue at the appropriate timepoints. The tissues were fixed in 4% paraformaldehyde (PFA; Thermo Fisher Scientific) for 24 h followed by storage in 0.1% PFA. The tissues were then embedded in agarose and submerged in de-ionized water for acoustic coupling prior to imaging. We performed 3D PAT/US imaging of the LCA using 1210 nm light for lipid-specific imaging and 1400 nm for off-resonant imaging. Ten frames were acquired at each location and then processed using median averaging to minimize PAT fluctuations and laser-induced noise.

**TABLE I.** Initial *ex vivo* PAT experiments. **⊗** denotes both euthanasia and PAT imaging. PAT, photoacoustic tomography; PCL, partial carotid ligation.

Purpose	Experimental groups	Sex	n	Imaging timepoints (weeks)					
				1	2	3	4	5	6
*Ex vivo* PAT lipid quantification validation	2 weeks post-PCL	Male	4		**⊗**				
	4 weeks post-PCL	Male	3				**⊗**		
	6 weeks post-PCL	Female	4						**⊗**

#### 2. PAT lipid burden quantification

All excised vessels were sectioned at multiple locations (n = 27) and stained with lipophilic Oil Red O (ORO). A thresholding algorithm was used to quantify the percent lipid composition from the histological sections, which was then compared to the PAT signal-to-noise ratio (SNR) at the same anatomical locations. Percent lipid composition was calculated using the green channel of the ORO histology images to discriminate lipid dense pockets from surrounding tissues. The area of these lipid dense pockets was then divided by the cross-sectional area of the carotid artery to quantify the lipid composition. The PAT SNR was obtained by measuring the lipid-specific signal intensity from the grayscale short-axis carotid artery image divided by a 16 × 16 pixel background region of interest. The location of the histological section was then matched to the location of the short-axis carotid artery PAT image. Finally, regression statistics were used to assess the goodness of fit between the lipid composition and PAT signal.

### E. Cross-sectional PAT and longitudinal ultrasound plaque characterization

#### 1. PAT and ultrasound image acquisition

*In vivo* hemodynamic, morphological, and kinematic changes, as well as *in situ* plaque lipid composition and volume, were quantified in both the LCA and right carotid arteries (RCA) using a small animal ultrasound system (Vevo 3100, FUJIFILM VisualSonics) and the PAT system described in Sec. V A. Table II contains a summary of all animal groups and imaging timepoints. The PCL procedure was performed on all animals at six weeks of age in combination with the administration of a Western diet. We used 1100 nm and 1210 nm for PAT hemoglobin and lipid characterization, respectively. The ultrasound imaging protocol included the acquisition of pulsed wave Doppler (PWD) for mean and peak antegrade and retrograde flow quantification, M-mode for green-langrage circumferential cyclic strain measurements, and respiratory-gated 4DUS for diameter and volume quantification, as well as maximum first-principal Green-Lagrange strain using a DDE method.^19^ The 4DUS data were acquired using standard acquisition, sharp processing style, frame rate of 300 Hz, and a step size of 0.05 mm. Moreover, five PWD and M-mode measurements were acquired between the carotid artery bifurcation and clavicle to track regional changes in vessel hemodynamics and kinematics. The mice were then euthanized at the study end point and perfused with saline and 1% agarose. The left and right common carotid artery were then exposed and imaged *in situ* using 3D PAT and ultrasound with 1100 nm, 1210 nm, and 1400 nm light for blood and lipid burden quantification as described in Sec. II D.

**TABLE II.** Animal groups and ultrasound imaging timepoints for plaque characterization. • denotes ultrasound timepoints, and “**⊗**” denotes ultrasound, PAT, and euthanize timepoints. PCL, partial carotid ligation; WD, Western diet.

Purpose	Experimental groups	n	Imaging timepoints (days)					
			0	1	4	7	10	14
Lipid deposition over time	Day 7 post-PCL +WD	8	•	•	•	**⊗**		
	Day 10 post-PCL +WD	8	•	•	•	•	**⊗**	
	Day 14 post-PCL +WD	8	•	•	•	•	•	**⊗**
	Sham control +WD	5	•	•	•	•	•	**⊗**
Strain predictor of lipid deposition	Day 4 post-PCL +WD	5	•	•	**⊗**			
	Day 4 post-PCL + chow	6	•	•	**⊗**			

#### 2. Hemodynamic and kinematic characterization

Mean and peak blood flow velocities were quantified in Vevo LAB (FUJIFILM, VisualSonics) by segmenting five pulsed wave waveforms at each location for all timepoints. A custom MATLAB (R2018a, Mathworks, Inc., Natick, MA) algorithm was used to execute a DDE method to calculate the Green-Lagrange strain as previously described.^18,19^ Briefly, we resampled the 4DUS dataset into 50 *μ*m^3^ isotropic voxels prior to DDE analysis of the Green-Lagrange strain. This algorithm utilized Gauss-Newton optimization to iteratively minimize voxel intensity differences between 3D volumes at successive timepoints by updating the warping function parameter. This warping function parameter spatially mapped the voxels between the undeformed volumes at timepoint t_1_ to deformed volumes at timepoint t_2_. Following optimization, the warping function is utilized to directly compute the deformation gradient tensor, which is then used to calculate the 3D maximum principal Green-Lagrange strain. The first maximum principal Green-Lagrange strain is then overlaid onto the solid carotid artery mesh to visualize the changes in pulsatility throughout the vessel. We also compared day 14 post-PCL DDE method derived strain measurements to M-mode derived circumferential cyclic strain measurements using Eq. (2). In this equation, D_sys_ and D_dia_ represent the vessel diameter at systole and diastole, respectively. Three distinct M-mode measurements were performed at five different regions along the day 14 post-PCL dataset

$$\epsilon =\frac{1}{2}\left[{\left(\frac{D_{\mathrm{sys}}}{D_{\mathrm{dia}}}\right)}^{2}-1\right]\;x\;100\%.$$ (2)

#### 3. Morphological characterization

Carotid artery diameters and volumes were quantified by extracting the systolic timepoints from the 4DUS data using the FSL toolbox (FMIRB, Oxford) and segmenting the lumen in SimVascular.^51^ Outer wall segmentations were not performed due to difficulty differentiating the artery from surrounding tissues. The segmentation path was created by placing control-points along both the LCA and RCA, followed by placing contours along the generated path to segment the lumen. Solid models of these 3D segmentations were exported as STL files and imported into Meshmixer (Autodesk Research, California) to calculate the lumen volumes. Additionally, these STL files were then imported into MATLAB to extract diameter measurements along the carotid artery to quantify regional morphological changes throughout the study.

#### 4. Hemoglobin localization and lipid deposition

A custom MATLAB script was utilized to quantify hemoglobin and lipid deposition and volume throughout the carotid artery. We performed quantitative analysis on all 1100 nm hemoglobin datasets that were confirmed to be successfully saline and agarose perfused using gross dissection images (supplementary material, Fig. S1). PAT-specific optical contrast was first quantified using a thresholding technique to discriminate PAT and background pixels in an approximately 7x3 mm region of interest around the carotid artery.^1^ Carotid arteries were then segmented from long-axis ultrasound images to ensure that the data analysis did not include contrast from extraneous tissues and image artifacts. We calculated the pixel-wise SNR by dividing the PAT-specific pixel intensity by the background noise that is measured using a 16 × 16 pixel region of interest. The hemoglobin and lipid-specific SNR was then averaged along the short-axis slices along the carotid artery to plot the lipid distribution throughout the vessel. The pixels associated with the PAT signal in this 3D dataset were also used to calculate lipid and hemoglobin volume, as shown in Eq. (3)

$$\text{Lipid}\;\text{Volume}\;={\;P}_{L}\times P_{W}\times P_{T}\times P_{L}.$$ (3)

Here, the pixel length (P_L_) and width (P_W_) are determined by user imputed calibration using the ultrasound scale bar, pixel thickness (P_T_) was set by the 0.193 mm elevational resolution of our ultrasound transducer, and the number of lipid-specific pixels (P_L_) was determined through the MATLAB script.

### F. Histology and immunohistochemistry

We dissected the LCA and RCA from the animal and fixed the tissue in 4% PFA (Thermo Fisher Scientific) for 24 h followed by placing the tissue in optimal cutting temperature compound (Thermo Fisher Scientific) and snap freezing for cryosectioning. The vessels were then processed for hematoxylin and eosin (H&E), Movat's pentachrome, and ORO histological staining, as well as F4/80 macrophage immunohistochemistry. Inflammatory burden was quantified by performing color deconvolution to calculate percent F4/80 stain with respect to the cross-sectional area of the histological section.

### G. Statistical analysis

All datasets were assessed using a normality test prior to statistical testing. Linear regression statistics were performed to evaluate the correlation between plaque lipid composition vs lipid specific PAT SNR. A one way analysis of variance (ANOVA) with a Tukey post-hoc test was used to determine statistical significance in *ex vivo* plaque lipid composition, macrophage infiltration, and histological outer diameter. Additionally, linear mixed-effects models statistical tests were performed on log-transformed mean and peak velocity, volume/length, diameter, strain, and compositional responses. Statistical significance was defined at *p < *0.05. We performed all statistical analyses in Statistical Analysis Software (SAS) and Prism 7 (GraphPad).

## SUPPLEMENTARY MATERIAL

See the supplementary material for gross surgical and dissection carotid artery images, magnified histology, animal weights, peak velocity and volume data, day 4 post-PCL strain, and histology data, as well as sham hemodynamic, kinematic, and morphological results. Additionally, data regarding DDE method comparison to circumferential cyclic strain analysis, and individual mouse hemoglobin and lipid specific PAT distribution can also be found in the supplementary material.

## References

[1] G. S. Sangha , E. H. Phillips , and C. J. Goergen , “ In vivo photoacoustic lipid imaging in mice using the second near-infrared window,” Biomed. Opt. Express 8, 736–742 (2017). 10.1364/BOE.8.000736 28270980 PMC5330553

[2] E. J. Benjamin , M. J. Blaha , S. E. Chiuve , M. Cushman , S. R. Das *et al.*, “ Heart disease and stroke statistics—2017 update: A report from the American Heart Association,” Circulation 135, e146–e603 (2017). 10.1161/CIR.0000000000000485 28122885 PMC5408160

[3] C. V. Bourantas , H. M. Garcia-Garcia , K. K. Naka , A. Sakellarios , L. Athanasiou *et al.*, “ Hybrid intravascular imaging: Current applications and prospective potential in the study of coronary atherosclerosis,” J. Am. Coll. Cardiol. 61, 1369–1378 (2013). 10.1016/j.jacc.2012.10.057 23500282

[4] C. V. Bourantas , F. A. Jaffer , F. J. Gijsen , G. Van Soest , S. P. Madden *et al.*, “ Hybrid intravascular imaging: Recent advances, technical considerations, and current applications in the study of plaque pathophysiology,” Eur. Heart J. 38, 400–412 (2017). 10.1093/eurheartj/ehw097 27118197 PMC5837589

[5] K. Jansen , G. van Soest , and A. F. van der Steen , “ Intravascular photoacoustic imaging: A new tool for vulnerable plaque identification,” Ultrasound Med. Biol. 40, 1037–1048 (2014). 10.1016/j.ultrasmedbio.2014.01.008 24631379

[6] K. Jansen , A. F. van der Steen , M. Wu , H. M. van Beusekom , G. Springeling *et al.*, “ Spectroscopic intravascular photoacoustic imaging of lipids in atherosclerosis,” J. Biomed. Opt. 19, 026006 (2014). 10.1117/1.JBO.19.2.026006 24522806

[7] B. Wang , A. Karpiouk , D. Yeager , J. Amirian , S. Litovsky *et al.*, “ Intravascular photoacoustic imaging of lipid in atherosclerotic plaques in the presence of luminal blood,” Opt. Lett. 37, 1244–1246 (2012). 10.1364/OL.37.001244 22466209

[8] D. E. Yeager , A. B. Karpiouk , B. Wang , J. H. Amirian , K. V. Sokolov *et al.*, “ Intravascular photoacoustic imaging of exogenously labeled atherosclerotic plaque through luminal blood,” J. Biomed. Opt. 17, 106016 (2012). 10.1117/1.JBO.17.10.106016 23224013 PMC3473229

[9] A. Kole , Y. Cao , J. Hui , I. A. Bolad , M. Alloosh *et al.*, “ Comparative quantification of arterial lipid by intravascular photoacoustic-ultrasound imaging and near-infrared spectroscopy-intravascular ultrasound,” J. Cardiovasc. Transl. Res. 12, 211–220 (2019). 10.1007/s12265-018-9849-2 30488332 PMC6611754

[10] E. I. Galanzha , M. Sarimollaoglu , D. A. Nedosekin , S. G. Keyrouz , J. L. Mehta , and V. P. Zharov , “ In vivo flow cytometry of circulating clots using negative photothermal and photoacoustic contrasts,” Cytometry, Part A 79, 814–824 (2011). 10.1002/cyto.a.21106 PMC3366468 21976458

[11] H. J. Jawad , M. Sarimollaoglu , A. S. Biris , and V. P. Zharov , “ Dynamic blood flow phantom with negative and positive photoacoustic contrasts,” Biomed. Opt. Express 9, 4702–4713 (2018). 10.1364/BOE.9.004702 30319897 PMC6179420

[12] N. Dana , L. Di Biase , A. Natale , S. Emelianov , and R. Bouchard , “ In vitro photoacoustic visualization of myocardial ablation lesions,” Heart Rhythm 11, 150–157 (2014). 10.1016/j.hrthm.2013.09.071 24080065 PMC4007201

[13] M. Wright , E. Harks , S. Deladi , F. Suijver , M. Barley *et al.*, “ Real-time lesion assessment using a novel combined ultrasound and radiofrequency ablation catheter,” Heart Rhythm 8, 304–312 (2011). 10.1016/j.hrthm.2010.10.039 21044698

[14] S. Iskander-Rizk , P. Kruizinga , A. F. Van Der Steen , and G. van Soest , “ Spectroscopic photoacoustic imaging of radiofrequency ablation in the left atrium,” Biomed. Opt. Express 9, 1309–1322 (2018). 10.1364/BOE.9.001309 29541523 PMC5846533

[15] Y. Cao , A. Kole , J. Hui , Y. Zhang , J. Mai *et al.*, “ Fast assessment of lipid content in arteries in vivo by intravascular photoacoustic tomography,” Sci. Rep. 8, 2400 (2018). 10.1038/s41598-018-20881-5 29402963 PMC5799328

[16] M. Wu , K. Jansen , A. F. W. van der Steen , and G. van Soest , “ Specific imaging of atherosclerotic plaque lipids with two-wavelength intravascular photoacoustics,” Biomed. Opt. Express 6, 3276–3286 (2015). 10.1364/BOE.6.003276 26417500 PMC4574656

[17] A. H. Soepriatna , A. K. Yeh , A. D. Clifford , S. E. Bezci , G. D. O'Connell , and C. J. Goergen , “ Three-dimensional myocardial strain correlates with murine left ventricular remodelling severity post-infarction,” J. R. Soc. Interface 16, 20190570 (2019). 10.1098/rsif.2019.0570 31744418 PMC6893492

[18] H. L. Cebull , A. H. Soepriatna , J. J. Boyle , S. M. Rothenberger , and C. J. Goergen , “ Strain mapping from four-dimensional ultrasound reveals complex remodeling in dissecting murine abdominal aortic aneurysms,” J. Biomech. Eng. 141, 060907 (2019). 10.1115/1.4043075 30840030

[19] J. J. Boyle , A. Soepriatna , F. Damen , R. A. Rowe , R. B. Pless *et al.*, “ Regularization-free strain mapping in three dimensions, with application to cardiac ultrasound,” J. Biomech. Eng. 141, 011010 (2019). 10.1115/1.4041576 PMC6298532 30267039

[20] B. Paigen , A. Morrow , P. A. Holmes , D. Mitchell , and R. A. Williams , “ Quantitative assessment of atherosclerotic lesions in mice,” Atherosclerosis 68, 231–240 (1987). 10.1016/0021-9150(87)90202-4 3426656

[21] C. Cheng , D. Tempel , R. Van Haperen , A. Van Der Baan , F. Grosveld *et al.*, “ Atherosclerotic lesion size and vulnerability are determined by patterns of fluid shear stress,” Circulation 113, 2744–2753 (2006). 10.1161/CIRCULATIONAHA.105.590018 16754802

[22] J. J. Khatri , C. Johnson , R. Magid , S. M. Lessner , K. M. Laude *et al.*, “ Vascular oxidant stress enhances progression and angiogenesis of experimental atheroma,” Circulation 109, 520–525 (2004). 10.1161/01.CIR.0000109698.70638.2B 14744973

[23] A. Kumar and V. Lindner , “ Remodeling with neointima formation in the mouse carotid artery after cessation of blood flow,” Arterioscler., Thrombosis, Vasc. Biol. 17, 2238–2244 (1997). 10.1161/01.ATV.17.10.2238 9351395

[24] D. Nam , C.-W. Ni , A. Rezvan , J. Suo , K. Budzyn *et al.*, “ Partial carotid ligation is a model of acutely induced disturbed flow, leading to rapid endothelial dysfunction and atherosclerosis,” Am. J. Physiol. 297, H1535–H1543 (2009). 10.1152/ajpheart.00510.2009 PMC2770764 19684185

[25] Y.-M. Go , C. W. Kim , D. I. Walker , D. W. Kang , S. Kumar *et al.*, “ Disturbed flow induces systemic changes in metabolites in mouse plasma: A metabolomics study using ApoE^−/−^ mice with partial carotid ligation,” Am. J. Physiol. 308, R62–R72 (2015). 10.1152/ajpregu.00278.2014 PMC4281678 25377480

[26] C.-W. Ni , H. Qiu , A. Rezvan , K. Kwon , D. Nam *et al.*, “ Discovery of novel mechanosensitive genes in vivo using mouse carotid artery endothelium exposed to disturbed flow,” Blood 116, e66–e73 (2010). 10.1182/blood-2010-04-278192 20551377 PMC2974596

[27] H. Merino , S. Parthasarathy , and D. K. Singla , “ Partial ligation-induced carotid artery occlusion induces leukocyte recruitment and lipid accumulation—A shear stress model of atherosclerosis,” Mol. Cell. Biochem. 372, 267–273 (2013). 10.1007/s11010-012-1468-7 23054191

[28] V. A. Korshunov , H. Wang , R. Ahmed , D. M. Mickelsen , Q. Zhou *et al.*, “ Model-based vascular elastography improves the detection of flow-induced carotid artery remodeling in mice,” Sci. Rep. 7, 12081 (2017). 10.1038/s41598-017-12321-7 28935983 PMC5608712

[29] J. Soulis , G. Giannoglou , M. Dimitrakopoulou , V. Papaioannou , S. Logothetides , and D. Mikhailidis , “ Influence of oscillating flow on LDL transport and wall shear stress in the normal aortic arch,” Open Cardiovasc. Med. J. 3, 128 (2009). 10.2174/1874192400903010128 19834577 PMC2761669

[30] S. Glagov , E. Weisenberg , C. K. Zarins , R. Stankunavicius , and G. J. Kolettis , “ Compensatory enlargement of human atherosclerotic coronary arteries,” New England J. Med. 316, 1371–1375 (1987). 10.1056/NEJM198705283162204 3574413

[31] S. Bonthu , D. D. Heistad , D. A. Chappell , K. G. Lamping , and F. M. Faraci , “ Atherosclerosis, vascular remodeling, and impairment of endothelium-dependent relaxation in genetically altered hyperlipidemic mice,” Arterioscler., Thromb., Vasc. Biol. 17, 2333–2340 (1997). 10.1161/01.ATV.17.11.2333 9409199

[32] V. A. Korshunov and B. C. Berk , “ Strain-dependent vascular remodeling: The “Glagov phenomenon” is genetically determined,” Circulation 110, 220–226 (2004). 10.1161/01.CIR.0000134958.88379.2E 15226209

[33] V. A. Korshunov , S. M. Schwartz , and B. C. Berk , “ Vascular remodeling: Hemodynamic and biochemical mechanisms underlying Glagov's phenomenon,” Arterioscler., Thromb., Vasc. Biol. 27, 1722–1728 (2007). 10.1161/ATVBAHA.106.129254 17541029

[34] R. Mitra , J. Qiao , S. Madhavan , G. L. O'Neil , B. Ritchie *et al.*, “ The comparative effects of high fat diet or disturbed blood flow on glycocalyx integrity and vascular inflammation,” Transl. Med. Commun. 3, 10 (2018). 10.1186/s41231-018-0029-9 30957020 PMC6447085

[35] J. Ternacle , F. Wan , D. Sawaki , M. Surenaud , M. Pini *et al.*, “ Short-term high-fat diet compromises myocardial function: A radial strain rate imaging study,” Eur. Heart J. 18, 1283–1291 (2017). 10.1093/ehjci/jew316 28062567

[36] G. S. Sangha , A. Busch , A. Acuna , A. G. Berman , E. H. Phillips *et al.*, “ Effects of iliac stenosis on abdominal aortic aneurysm formation in mice and humans,” J. Vasc. Res. 56, 217–229 (2019). 10.1159/000501312 31272099 PMC6819217

[37] S. Wang , J. Lin , T. Wang , X. Chen , and P. Huang , “ Recent advances in photoacoustic imaging for deep-tissue biomedical applications,” Theranostics 6, 2394 (2016). 10.7150/thno.16715 27877243 PMC5118603

[38] G. S. Sangha and C. J. Goergen , “ Photoacoustic tomography: Applications for atherosclerosis imaging,” J. Opt. 18, 084005 (2016). 10.1088/2040-8978/18/8/084005

[39] L. V. Wang and J. Yao , “ A practical guide to photoacoustic tomography in the life sciences,” Nat. Methods 13, 627 (2016). 10.1038/nmeth.3925 27467726 PMC4980387

[40] I. J. Shin , S.-M. Shon , D. Schellingerhout , J.-Y. Park , J.-Y. Kim *et al.*, “ Characterization of partial ligation-induced carotid atherosclerosis model using dual-modality molecular imaging in ApoE knock-out mice,” PLoS One 8, e73451 (2013). 10.1371/journal.pone.0073451 24069197 PMC3772018

[41] A. Millon , M. Sigovan , L. Boussel , J.-L. Mathevet , V. Louzier *et al.*, “ Low WSS induces intimal thickening, while large WSS variation and inflammation induce medial thinning, in an animal model of atherosclerosis,” PLoS One 10, e0141880 (2015). 10.1371/journal.pone.0141880 26575029 PMC4648591

[42] C. A. Foss , D. Bedja , R. C. Mease , H. Wang , D. A. Kass *et al.*, “ Molecular imaging of inflammation in the ApoE-/-mouse model of atherosclerosis with IodoDPA,” Biochem. Biophys. Res. Commun. 461, 70–75 (2015). 10.1016/j.bbrc.2015.03.171 25858322 PMC4426030

[43] W. Yu , J. C. Braz , A. M. Dutton , P. Prusakov , and M. Rekhter , “ In vivo imaging of atherosclerotic plaques in apolipoprotein E deficient mice using nonlinear microscopy,” J. Biomed. Opt. 12, 054008 (2007). 10.1117/1.2800337 17994896

[44] A. Kharine , S. Manohar , R. Seeton , R. G. M. Kolkman , R. A. Bolt *et al.*, “ Poly (vinyl alcohol) gels for use as tissue phantoms in photoacoustic mammography,” Phys. Med. Biol. 48, 357 (2003). 10.1088/0031-9155/48/3/306 12608612

[45] A. Seki , K. Iwai , T. Katagiri , and Y. Matsuura , “ Forward-viewing photoacoustic imaging probe with bundled ultra-thin hollow optical fibers,” J. Opt. 18, 074015 (2016). 10.1088/2040-8978/18/7/074015

[46] D. Cai , Z. Li , and S.-L. Chen , “ In vivo deconvolution acoustic-resolution photoacoustic microscopy in three dimensions,” Biomed. Opt. Express 7, 369–380 (2016). 10.1364/BOE.7.000369 26977346 PMC4771455

[47] K. Xiong , S. Yang , X. Li , and D. Xing , “ Autofocusing optical-resolution photoacoustic endoscopy,” Opt. Lett. 43, 1846–1849 (2018). 10.1364/OL.43.001846 29652380

[48] G. S. Sangha , N. J. Hale , and C. J. Goergen , “ Adjustable photoacoustic tomography probe improves light delivery and image quality,” Photoacoustics 12, 6–13 (2018). 10.1016/j.pacs.2018.08.002 30175045 PMC6118042

[49] R. Li , E. Phillips , P. Wang , C. J. Goergen , and J. X. Cheng , “ Label‐free in vivo imaging of peripheral nerve by multispectral photoacoustic tomography,” J. Biophotonics 9, 124–128 (2016). 10.1002/jbio.201500004 25904317

[50] F. W. Damen , A. R. Adelsperger , K. E. Wilson , and C. J. Goergen , “ Comparison of traditional and integrated digital anesthetic vaporizers,” J. Am. Assoc. Lab. Animal Sci. 54, 756–762 (2015). PMC4671791 26632785

[51] A. Updegrove , N. M. Wilson , J. Merkow , H. Lan , A. L. Marsden , and S. C. Shadden , “ SimVascular: An open source pipeline for cardiovascular simulation,” Ann. Biomed. Eng. 45, 525–541 (2017). 10.1007/s10439-016-1762-8 27933407 PMC6546171

